# Kidney function and acid–base status as gatekeepers of natriuretic and diuretic responsiveness in chronic HF: insights from the DEA-HF clinical trial

**DOI:** 10.1093/ehjcvp/pvag019

**Published:** 2026-05-18

**Authors:** Ina Volis, Amit Gruber, Aharon (Ronnie) Abbo, Doron Aronson, Nicolas Girerd, Søren Lund Kristensen, Robert Zukermann, Natalia Alberkant, Elena Sitnitsky, Anton Kruger, Polina Khasis, Evgeny Bravo, Boaz Elad, Ludmila Helmer Levin, Oren Caspi

**Affiliations:** The Heart Failure Unit, Department of Cardiology, Rambam Health Care Campus, Haifa 3109601, Israel; The Heart Failure Unit, Department of Cardiology, Rambam Health Care Campus, Haifa 3109601, Israel; The Ruth and Bruce Rappaport Faculty of Medicine, Technion, Haifa 3525433, Israel; The Heart Failure Unit, Department of Cardiology, Rambam Health Care Campus, Haifa 3109601, Israel; The Ruth and Bruce Rappaport Faculty of Medicine, Technion, Haifa 3525433, Israel; The Heart Failure Unit, Department of Cardiology, Rambam Health Care Campus, Haifa 3109601, Israel; The Ruth and Bruce Rappaport Faculty of Medicine, Technion, Haifa 3525433, Israel; Université de Lorraine, INSERM, Centre d'Investigations Cliniques-Plurithématique 1433, CHRU Nancy, and INSERM U1116, CHRU, F-CRIN INI-CRCT (Cardiovascular and Renal Clinical Trialists), Nancy 54000, France; Department of Cardiology, Copenhagen University Hospital—Rigshospitalet, Copenhagen 2100, Denmark; The Heart Failure Unit, Department of Cardiology, Rambam Health Care Campus, Haifa 3109601, Israel; The Heart Failure Unit, Department of Cardiology, Rambam Health Care Campus, Haifa 3109601, Israel; The Heart Failure Unit, Department of Cardiology, Rambam Health Care Campus, Haifa 3109601, Israel; The Heart Failure Unit, Department of Cardiology, Rambam Health Care Campus, Haifa 3109601, Israel; The Heart Failure Unit, Department of Cardiology, Rambam Health Care Campus, Haifa 3109601, Israel; The Heart Failure Unit, Department of Cardiology, Rambam Health Care Campus, Haifa 3109601, Israel; The Heart Failure Unit, Department of Cardiology, Rambam Health Care Campus, Haifa 3109601, Israel; The Heart Failure Unit, Department of Cardiology, Rambam Health Care Campus, Haifa 3109601, Israel; The Heart Failure Unit, Department of Cardiology, Rambam Health Care Campus, Haifa 3109601, Israel; The Ruth and Bruce Rappaport Faculty of Medicine, Technion, Haifa 3525433, Israel; Clinical Research Institute at Rambam (CRIR), Haifa 3109601, Israel

**Keywords:** Heart failure, Chronic renal disease, Diuretic resistance, Serum bicarbonate, Diuretics, Natriuresis

## Abstract

**Aims:**

In chronic heart failure (HF), decongestion is hindered by reduced renal function and diuretic resistance. This study compared the effect of eGFR and serum bicarbonate on three diuretic regimens in congestion-refractory HF patients.

**Methods and results:**

This is a prespecified post-hoc analysis of DEA-HF, a randomized, crossover trial (*n* = 42). Patients received, in random order, weekly treatments of IV furosemide 250 mg; oral metolazone 5mg + IV furosemide 250 mg; and IV acetazolamide 500mg + IV furosemide 250 mg. Primary endpoint: 6h-Natriuresis; secondary: 6h-diuresis and decongestion measures at 7 ± 3 days. Mixed models assessed effect modification by eGFR (≤30 vs. > 30 mL/min/1.73m^2^) and by serum bicarbonate (≤vs.> median-29.6 mmol/L). Higher eGFR was associated with greater natriuresis and diuresis (4735 mg vs. 3211 mg, *P* = 0.0004; 1.93L vs. 1.49L, *P* = 0.0078). In patients with eGFR > 30, addition of metolazone to furosemide led to higher natriuresis compared to acetazolamide addition (5525 mg vs. 4379 mg, *P* = 0.04) or to furosemide monotherapy (5525 mg vs. 4303 mg, *P* = 0.014); no regimen differences were observed at eGFR ≤ 30. Independently, higher serum bicarbonate predicted greater natriuresis and diuresis (4858 mg vs. 3576 mg, *P* = 0.0008; 1.99 vs. 1.56L, *P* = 0.0014). There was no difference in clinical decongestion measures. All regimens were well-tolerated with comparable safety concerns regarding renal function, electrolyte disturbances, or hypotension.

**Conclusion:**

In ambulatory patients with congestion-refractory HF and eGFR > 30 mL/min/1.73m^2^, natriuretic and diuretic responses are augmented across all regimens, with metolazone providing additional improvement in natriuresis. When eGFR ≤ 30 mL/min/1.73m^2^, neither metolazone nor acetazolamide provides additional benefit. High serum bicarbonate predicts better natriuretic and diuretic response, as well as additional benefit from metolazone treatment. High-dose diuretics had comparable safety profiles across the eGFR spectrum.

## Graphical Abstract

**Graphical Abstract:**
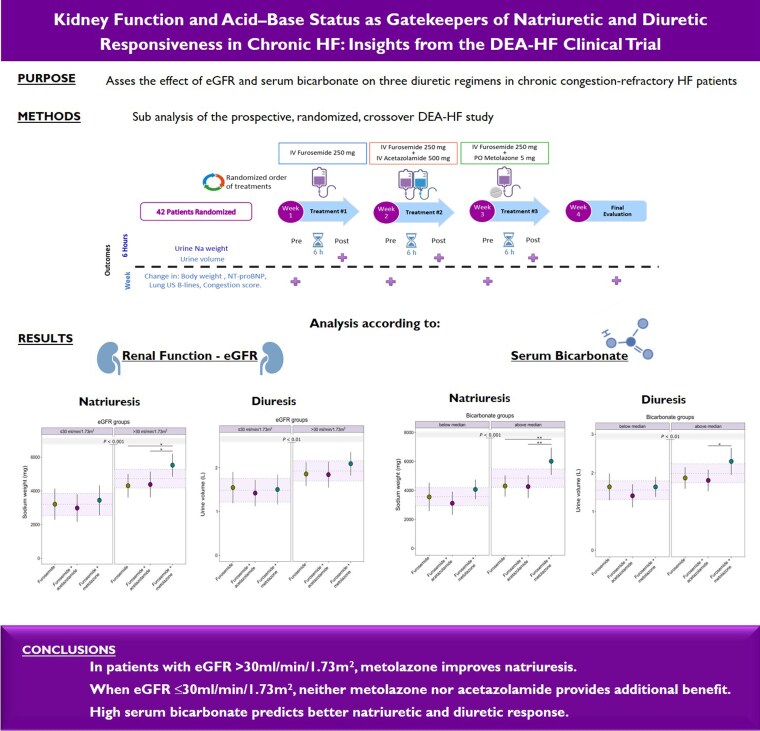

## Introduction

Diuresis is the cornerstone of treatment in both acute and chronic symptomatic patients with heart failure (HF).^1–3^ Patients with chronic HF often have coexisting chronic kidney disease (CKD), with a reported prevalence of CKD ranging from 50% to 70% among patients with HF.^4,5^ Achieving effective decongestion and diuresis in HF patients with CKD is an arduous task frequently complicated by diuretic resistance and a high incidence of worsening renal function (WRF) and refractory congestion.^4,6^ The efficiency of natriuresis is influenced by many factors, including type and dosage of diuretic agents, the extent of volume overload, acid–base balance, and, importantly, renal function,^6–8^ which is commonly represented by the estimated glomerular filtration rate (eGFR),^6^ which ultimately determines the overall diuretic response and the success of decongestion.

A widely adopted strategy to circumvent diuretic resistance is sequential nephron blockade, in which loop diuretics are combined with adjunctive agents (e.g. thiazide-like diuretics or acetazolamide) so as to inhibit sodium reabsorption at multiple sites within the nephron, thereby potentiating natriuresis and diuresis.^7–10^ While the effects of individual diuretics across various eGFR ranges have been studied,^11–17^ the net effect of diuretic combinations over the full spectrum of renal function remains elusive. The complexity arises from nonlinear interactions among transport segments, compensatory renal adaptation, and altered drug handling in renal impairment.^18^ Furthermore, much of the extant evidence on cardio-renal interactions and the utility of sequential nephron blockade derives from investigations in the context of acute decompensated HF. In contrast, there is a notable paucity of data guiding the use of combination diuretic therapy in patients with chronic HF, where long-term remodeling, neurohormonal adaptation, and evolving renal function may modulate both efficacy and safety.

Loop diuretics remain the cornerstone of decongestive therapy. Although combination diuretic therapies are frequently employed in the management of chronic HF, particularly in patients with concomitant CKD, their efficacy and safety have not been systematically assessed.

Recent data shed light on the interaction between combination diuretic therapies and kidney function in the setting of acute decompensated HF.^17,19^ These studies provide divergent insights regarding the effectiveness of combination therapies in patients with low eGFR. While thiazides’ decongestive effect is more pronounced in patients with higher eGFR, acetazolamide was found effective across the entire eGFR spectrum; its natriuretic and diuretic effects were proportionally greater in patients with lower baseline renal function^12^ and a higher level of serum bicarbonate (a marker of neurohormonal activation).^20^ For patients with significantly reduced eGFR, some have suggested that adding metolazone may enhance natriuresis by overcoming distal tubular sodium reabsorption^7,21^; however, robust data supporting this strategy in the chronic HF setting are lacking. These findings raise an important clinical question: Does the same relationship between renal function, bicarbonate levels, and diuretic efficacy hold true in patients with chronic HF?

Accordingly, we conducted a pre-specified post-hoc analysis of the Heart Failure Diuresis Efficacy Comparison (DEA-HF) randomized crossover trial, which compared -during weekly ambulatory diuresis sessions - three commonly used regimens: intravenous (IV) furosemide alone, IV furosemide plus oral metolazone, and IV furosemide plus IV acetazolamide.^1^ Leveraging the trial’s within-patient design and natriuresis-centered endpoints, we evaluated how eGFR and baseline serum bicarbonate modify the natriuretic and diuretic responses to each of the three different regimens, in ambulatory patients with chronic HF and refractory congestion. The overarching aim was to delineate regimen-by-renal-function interactions and identify patient-level predictors of diuretic responsiveness that could inform the rational selection of sequential nephron-blockade strategies in the ambulatory setting.

## Methods

### Patient population

The DEA-HF was a prospective, investigator-initiated randomized controlled trial, carried out at a single ambulatory HF day-care center. The study was originally designed to compare the efficacy of three common diuretic regimens in patients with chronic HF and refractory volume overload.^1^ Patients included in the study had a confirmed diagnosis of HF and at least one clinical sign of congestion. Participants had been prescribed at least two guideline-directed medical therapy drugs along with oral diuretic treatment for a minimum of 30 days prior to randomization. Patients were excluded if they had a baseline eGFR below 20 mL/min/1.73 m^2^, a mean blood pressure below 60 mmHg, or had received IV inotropes, vasopressors, or nitroprusside within 14 days prior to randomization. A total of 42 patients were included in the study. The study was approved by the institutional review board and registered on ClinicalTrials.gov (NCT05904808). All participants provided written informed consent.

### Trial design

**Figure 1:**
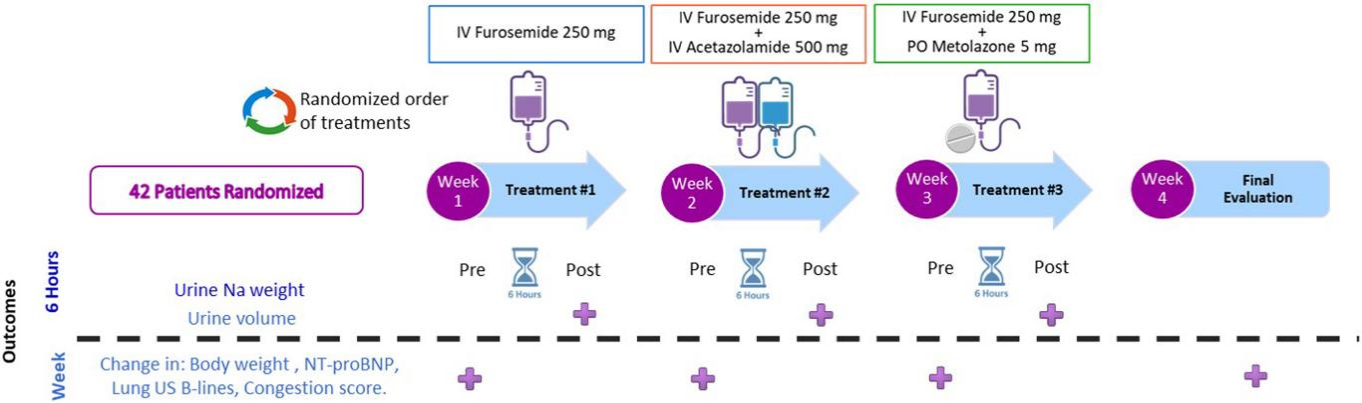
Overview of the DEA-HF study design. Schematic overview of the DEA-HF trial protocol. The primary outcome, natriuresis, was assessed 6 hours after each treatment. Secondary outcomes included diuresis (6-hour assessment) and body weight, serum NT-proBNP, lung ultrasound B-lines, and clinical congestion score, evaluated before each treatment.

The DEA-HF was designed as an open-label crossover study. All patients were planned to receive all three diuretic regimens tested. The order of administration was determined by an initial randomization, with one of six possible sequences. Randomization was stratified by left ventricular ejection fraction (LVEF, ≤50% or >50%) and patient gender. The study was conducted in the setting of an ambulatory day-care unit. Weekly treatments were scheduled (7 ± 3 days) with treatment duration of 4–5 hours. The diuretic regimens in the trial included (i) IV furosemide 250 mg, (ii) oral metolazone 5 mg, followed by IV furosemide 250 mg, and (iii) IV 500 mg acetazolamide, followed by IV furosemide 250 and 600 mg oral Magnesium-Diasporal. Furosemide was administered as a 40 mg IV bolus, followed by 210 mg in a 100 mL normal saline solution over 4 hours. Acetazolamide was administered in a 100 mL normal saline solution over 30–60 min. Patients were evaluated, and blood samples were collected weekly, prior to the administration of each treatment and on the final follow-up on week 4 (*Figure 1* describes study design).

In this sub-analysis, treatment effects were evaluated according to pretreatment eGFR and serum bicarbonate levels. Patients were categorized by eGFR (≤30 vs. > 30 mL/min/1.73 m^2^) and by serum bicarbonate (≤ vs. > median value of the study’s cohort, 29.6 mmol/L), as assessed prior to each treatment course. The effect of each diuretic regimen was analyzed in relation to the corresponding pretreatment eGFR and serum bicarbonate levels.

### Study endpoints

In line with the original DEA-HF, in the current sub-analysis, the primary outcome was total sodium excretion as measured in milligrams at 6 hours after treatment initiation (=spot urine concertation × urine volume). The secondary outcomes were total urine volume at 6 hours after treatment initiation and measures of decongestion that were evaluated before and 7 ± 3 days after each treatment: change in body weight, NT-ProBNP ratio, change in lung ultrasound (LUS) B-line count, and change in congestion score. The congestion score was comprised of pedal oedema, pleural effusion, and ascites, as previously described.^1^

Safety outcomes were as follows: (i) WRF, defined as an increase in serum creatinine >0.3 mg/dL or a decrease >30% in eGFR, calculated using the 2021 CKD-EPI formula^6^; (ii) hyponatremia, defined as serum sodium <135 mmol/dL; (iii) dyskalaemia, defined as serum potassium (K) < 3.5 mmol/dL or K > 5.6 mmol/dL; (iv) metabolic acidosis necessitating bicarbonate supplementation within 1 week of treatment; and (v) symptomatic hypotension. Safety outcomes were assessed within 1 week of the index treatment. Hospitalization for any reason terminated patient participation in the study.

### Statistical analysis

Linear mixed models followed by likelihood ratio tests were used to evaluate the effect of eGFR or serum bicarbonate on the different outcomes. Pretreatment eGFR was categorized into one of two groups: equal or lower than 30 mL/min/1.73 m^2^, or greater than 30 mL/min/1.73 m^2^. The eGFR threshold of 30 mL/min/1.73 m^2^ was selected *a priori* based on established CKD staging, distinguishing moderate from severe renal impairment and reflecting a clinically relevant cutoff in HF patient population. Pretreatment serum bicarbonate was categorized into either equal/lower than the median bicarbonate value, or above it. Patient identity was defined as a random effect, while fixed effects included the eGFR/bicarbonate category, treatment regimen, HF type (reduced or preserved LVEF), randomization group, and treatment timing (treatment given on visits 1, 2, or 3).

Post-hoc Bonferroni test was used to perform comparisons whenever overall differences were detected. The results are reported as point estimates with 95% confidence intervals (CI).

Differences in adverse events (AE) among the different eGFR groups were evaluated by fitting generalized linear mixed models for each predefined AE, followed by likelihood ratio tests.

All statistical tests were two-sided, and a *P* value of less than 0.05 was considered significant. Analyses were performed with the use of ‘lme4,’ ‘lmerTest,’ ‘ggplot2,’ ‘emmeans,’ and ‘gtsummary’ packages in R software version 4.3.0, and Stata software version 17.

## Results

### Baseline characteristics

Upon completion of the DEA-HF study,^1^ further data analyses were conducted to evaluate the impact of eGFR on the efficacy and safety on high-intensity IV diuretic regimens. The analysis included a cohort of 42 patients and 116 treatments administered in total. To evaluate the impact of eGFR, data from the three treatment visits (i.e. visit numbers 1, 2, or 3) was used to compare the impact of each diuretic regimen while stratified according to eGFR (≤/> 30 mL/min/1.73 m^2^).

The cohort consisted of clinically stable HF patients with chronic volume overload. There were 16 patients who received 46 treatments in the eGFR ≤30 mL/min/1.73 m^2^ group and 26 patients with 70 treatments in the eGFR >30 mL/min/1.73 m^2^ group.

The mean eGFR in the group with eGFR ≤30 mL/min/1.73 m^2^ was 24 ± 3 mL/min/1.73 m^2^, compared to 51 ± 17 mL/min/1.73 m^2^ in those with eGFR >30 mL/min/1.73 m^2^ (*P* < 0.001). Corresponding mean serum creatinine levels were 2.3 and 1.5 mg/dL, respectively (*P* < 0.001). The mean age (76 ± 7 vs. 70 ± 10), LVEF (42 ± 19 vs. 40 ± 22), and type of HF (56% vs. 46% with LVEF > 50%) were similar in both groups. Among participants with eGFR ≤30 mL/min/1.73 m^2^, there was a lower proportion of males (31% vs. 77%, *P* = 0.003). Patients in both eGFR groups had similar averaged vital signs (heart rate and blood pressure), weight, congestion scores, and New York Heart Association (NYHA) classification as well as mean dosage of baseline oral furosemide (*Table 1*). Patients with eGFR ≤30 mL/min/1.73 m^2^ had a significantly higher median serum NT-proBNP levels (8269 vs. 2980 pg/mL, *P* = 0.014) and lower haemoglobin levels (11.8 vs. 13.3 g/dL, *P* = 0.019) with a non-significant difference in ferritin and transferring saturation values and were less likely to be treated with renin-angiotensin-aldosterone system (RAAS) inhibitors. Notably, the vast majority of patients were receiving contemporary guideline-directed medical therapy, including SGLT2 inhibitors (100% in the eGFR ≤30 group and 92% in the eGFR >30 group) and mineralocorticoid receptor antagonists (88% and 92%, respectively).

**Table 1:** Patient characteristics

	eGFR Group (mL/min/1.73 m^2^)		
	≤30 (*n* = 16)^1^	>30 (*n* = 26)^1^	*P*-value^2^
**Characteristic**			
eGFR, mean ± STd (mL/min/1.73 m^2^)	24 ± 3	51 ± 17	<0.001
Age, years ± SD	76 ± 7	70 ± 10	0.071
Male sex, *n* (%)	5 (31%)	20 (77%)	0.003
LVEF, mean ± SD (%)	42 ± 19	40 ± 22	0.7
LVEF > 50%, *n* (%)	9 (56%)	12 (46%)	0.5
LVEF in HFpEF group, mean ± SD (%)	59 ± 4	61 ± 8	0.5
LVEF in HFrEF group, mean ± SD (%)	24 ± 8	21 ± 8	0.4
Heart rate, mean ± SD (beats/min)	69 ± 11	71 ± 14	0.7
Systolic BP, mean ± SD (mmHg)	119 ± 20	113 ± 13	0.4
Diastolic BP, mean ± SD (mmHg)	72 ± 10	69 ± 10	0.4
Weight, mean ± SD (kg)	84 ± 18	88 ± 18	0.5
Congestion parameters			
Congestion score—total points, median (IQR)	6 (4, 10)	6 (4, 8)	0.8
Ascites subscore (points), median (IQR)	0 (0, 2)	0 (0, 1)	0.2
Pleural effusion subscore (points), median (IQR)	0 (0, 0)	0 (0, 0)	0.3
Pedal edema subscore (points), median (IQR)	2 (1, 3)	1 (1, 2)	>0.9
Lung ultrasound B-lines count, median (IQR)	4 (2, 8)	4 (2, 11.5)	0.7
NYHA class			0.15
2, *n* (%)	0 (0%)	3 (12%)	
3, *n* (%)	11 (69%)	20 (77%)	
4, *n* (%)	5 (31%)	3 (12%)	
Laboratory			
NT-proBNP, median (IQR) (pg/mL)	8269 (3,244, 17,068)	2980 (1,422, 4106)	0.014
Serum haemoglobin (g/dL)	11.81 ± 1.85	13.27 ± 1.65	0.019
Serum sodium (mmol/liter)	139 ± 3	139 ± 3	0.6
Serum creatinine (mg/dL)	2.30 ± 0.47	1.51 ± 0.43	<0.001
Serum bicarbonate (mmol/liter)	29.0 ± 5.9	30.4 ± 3.9	0.4
Serum ferritin (ng/mL)	262 ± 242	142 ± 105	0.09
Transferrin saturation (%)	21 ± 9	20 ± 9	0.4
Comorbidities			
Ischemic aetiology, *n* (%)	10 (63%)	13 (50%)	0.4
History of atrial fibrillation, *n* (%)	14 (88%)	21 (81%)	0.7
Hypertension, *n* (%)	11 (69%)	22 (85%)	0.3
Diabetes, *n* (%)	13 (81%)	15 (58%)	0.12
Heart failure therapy			
Baseline furosemide dose (mg/day)	160 (160, 160)	140 (80, 160)	0.11
ACEi, ARB, or ARNI, *n* (%)	9 (56%)	23 (88%)	0.027
Beta blockers, *n* (%)	14 (100%)	24 (92%)	0.5
MRA, *n* (%)	14 (88%)	24 (92%)	0.6
SGLT2i, *n* (%)	16 (100%)	24 (92%)	0.5
Implantable cardioverter defibrillator, *n* (%)	1 (6.3%)	6 (24%)	0.2
Cardiac resynchronization therapy, *n* (%)	5 (31%)	5 (19%)	0.5
Pacemaker, *n* (%)	2 (13%)	3 (12%)	>0.9

^1^Mean ± SD; n/*n*(%); Median (IQR)

^2^Wilcoxon rank sum test; Fisher’s exact test

Abbreviations: ACEi, angiotensin-converting enzyme inhibitor; ARB, angiotensin II receptor blocker; ARNI, angiotensin receptor-neprilysin inhibitor; BP, blood pressure; eGFR, estimated glomerular filtration rate; HFpEF, heart failure with preserved ejection fraction; HFrEF, heart failure with reduced ejection fraction; LVEF, left ventricular ejection fraction; MRA, Mineralocorticoid receptor antagonist; NYHA, New York Heart Association; SD, standard deviation; SGLT2i, sodium–glucose cotransporter 2 inhibitor.

### Natriuresis and diuresis across eGFR and bicarbonate groups

**Figure 2:**
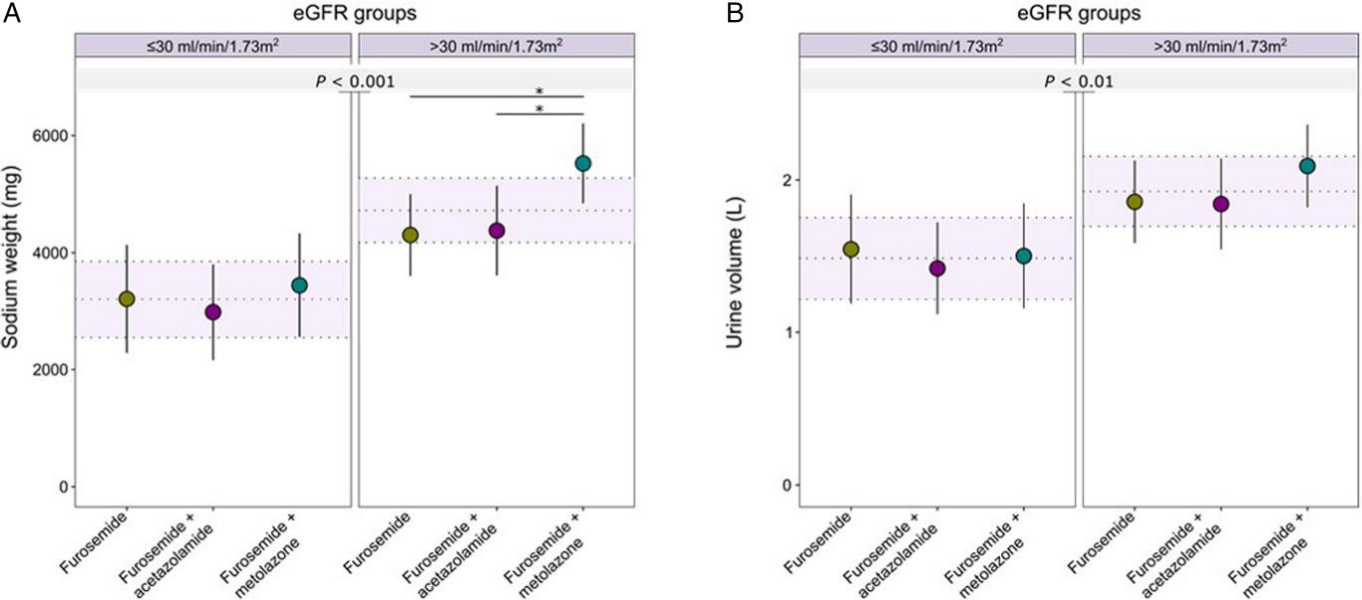
**Natriuresis and diuresis by eGFR category.** Total sodium excretion in mg; (urine sodium concentration × urine volume) (panel *A*) and total urine volume (panel *B*) were measured 6 h after treatment initiation. Higher eGFR correlated with significantly greater natriuretic and diuretic responses across all regimens (*P* < 0.001 and *P* < 0.01, respectively). Among patients with eGFR >30 mL/min/1.73 m^2^, metolazone was associated with higher natriuresis.

Seventy and 46 treatments in the eGFR above and below 30 mL/min/1.73 m^2^ were analyzed, respectively. An eGFR value above 30 mL/min/1.73 m^2^ resulted in a significantly greater natriuresis and diuresis, across all treatment groups (*Figures 2A* and *B*). The estimated mean natriuresis of all three treatment groups in the group with eGFR >30 mL/min/1.73 m^2^ was 4735 mg (95% CI: 4183–5228 mg), whereas in the group with eGFR ≤30 mL/min/1.73 m^2^, it was 3211 mg (95% CI: 2554–3868 mg, *P* = 0.0004). The estimated mean diuresis in the group with eGFR ≤30 mL/min/1.73 m^2^ was 1.49L (95% CI: 1.22–1.76L) compared to 1.93L (95% CI: 1.7–2.16L, *P* = 0.0078) in the group with eGFR >30 mL/min/1.73 m^2^ (*Figure 2B*).

When eGFR was above 30 mL/min/1.73 m^2^, addition of metolazone to furosemide led to a significantly greater natriuresis compared to the addition of acetazolamide to furosemide (5525 mg vs. 4379 mg, *P* = 0.04) or to furosemide administration alone (5525 mg vs. 4303 mg, *P* = 0.014), *Figure 2A*. With eGFR ≤30 mL/min/1.73 m^2^, natriuresis did not differ between treatment regimens.

**Figure 3:**
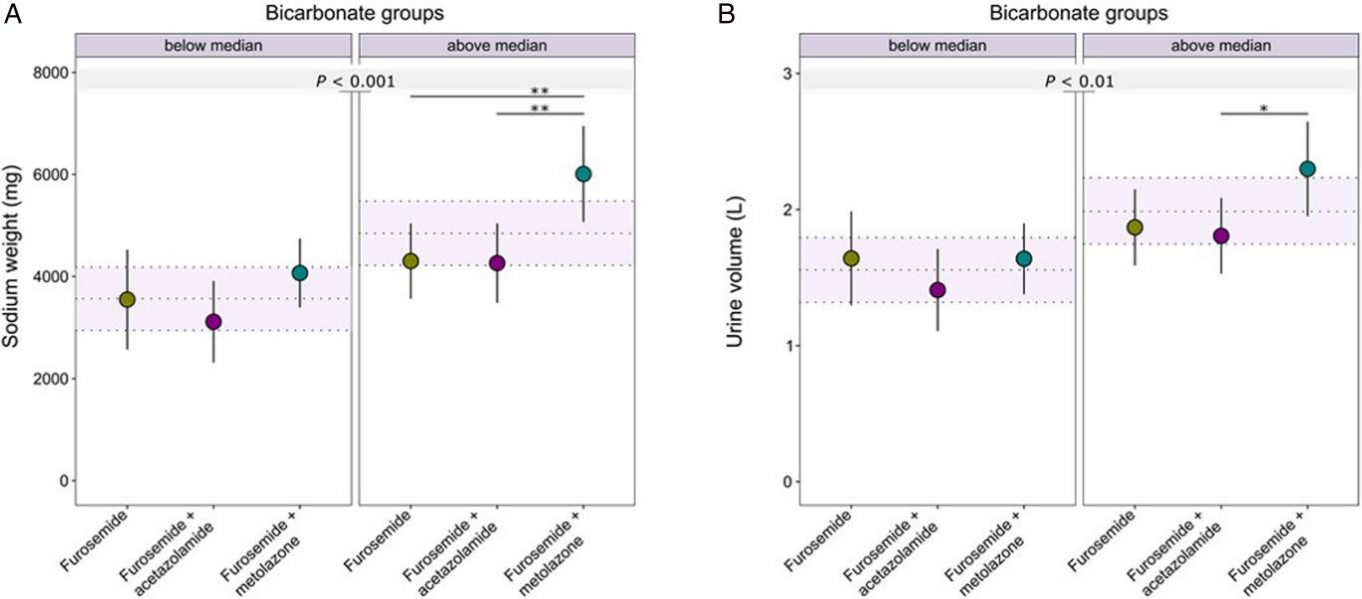
Natriuresis and diuresis by pretreatment serum bicarbonate levels. Total sodium excretion in mg (urine sodium concentration × urine volume) (panel *A*) and total urine volume (panel *B*) measured 6 h after treatment initiation, stratified by serum bicarbonate above or below the cohort median (=29.6 mmol/L). Higher serum bicarbonate was associated with greater natriuretic and diuretic responses across all regimens (*P* < 0.001 and *P* < 0.01, respectively). Among patients with higher bicarbonate, metolazone further enhanced natriuresis and diuresis.

The median serum bicarbonate level of the cohort was 29.6 mmol/L. Fifty-five treatments were analyzed when bicarbonate was higher than the median, and 55 treatments were below the cohort’s median. Higher than median pretreatment bicarbonate levels were also correlated with enhanced natriuretic and diuretic response (*Figures 3A* and *B*). This relationship was consistent across all three treatment regimens: furosemide, furosemide and acetazolamide, and furosemide and metolazone (Supplementary material online, *Figures S3A* and *S3B*).

In patients with lower than median pretreatment serum bicarbonate levels (range 19.1–29.6 mmol/L), the estimated average natriuresis was 3576 mg (95% CI: 2954–4199 mg). This was significantly lower than in those with higher than median serum bicarbonate levels (29.6–44.2 mmol/L), who had an average natriuresis of 4858 mg (95% CI: 4225–5490 mg; *P* = 0.0008). Adding metolazone to furosemide led to a significantly higher natriuresis (6008 mg, 95% CI: 5067–6950 mg) among patients with higher pretreatment serum bicarbonate levels compared to the addition of acetazolamide to furosemide (4262 mg, 95% CI: 3483–5042 mg, *P* = 0.0053) or to administration of furosemide solely (4302 mg, 95% CI: 3565–5039 mg, *P* = 0.0033), *Figure 3A*. Similarly, the average diuresis in patients with lower serum bicarbonate was 1.56L (95% CI: 1.32–1.80 L), compared to 1.99L (95% CI: 1.75–2.24 L; *P* = 0.0014) in patients with higher serum bicarbonate. Of the three treatments, in patients with higher than median pretreatment serum bicarbonate, the combination of furosemide with metolazone led to a significantly higher diuresis (2.3L, 95% CI: 1.95–2.65 L), compared to treatment with furosemide with acetazolamide (1.81L, 95%, CI: 1.53–2.08 L; *P* = 0.0314) but with similar diuretic impact compared to furosemide alone (1.87L, 95%, CI: 1.59–2.15; *P* = 0.065) (*Figure 3B*).

### Clinical outcomes across eGFR and bicarbonate groups

**Figure 4:**
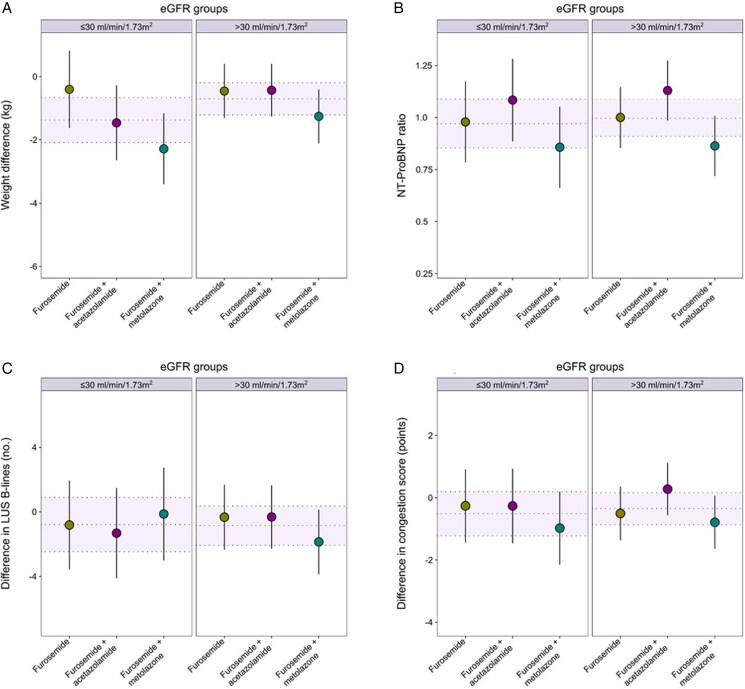
Clinical outcomes by eGFR group. Changes in body weight (panel *A*), NT-proBNP (panel *B*), lung ultrasound B-lines (panel *C*), and congestion score (panel *D*) after treatment, stratified by eGFR ≤30 and >30 mL/min/1.73 m^2^. Decongestion parameters did not differ between the groups.

There was no significant difference in decongestion parameters between the eGFR groups (*Figure 4*). In patients with eGFR >30 mL/min/1.73 m^2^, the mean change in body weight was −0.72 kg (95% CI: −1.23 to −0.20 kg), compared to −1.39 kg (95% CI: −2.10 to −0.67 kg) in the eGFR ≤30 mL/min/1.73 m^2^ group (*P* = 0.14, *Figure 4A*). The mean NT-proBNP ratio was 1 (95% CI: 0.90–1.10) in the higher eGFR group and 0.97 (95% CI: 0.85–1.10) in the lower eGFR group (*P* = 0.74, *Figure 4B*). The estimated mean reduction in LUS B-line count was −0.84 (95% CI: −2.05 to 0.38) in the higher eGFR group, compared to −0.75 (95% CI: −2.45 to 0.94) in the lower eGFR group (*P* = 0.94, *Figure 4C*). The estimated change in clinical congestion score was −0.34 (95% CI: −0.86 to 0.18) in the higher eGFR group and −0.50 (95% CI: −1.22 to 0.22) in the lower eGFR group (*P* = 0.72, *Figure 4D*). Similar outcomes in decongestion parameters were observed when stratifying patients by median pretreatment serum bicarbonate levels (Supplementary material online, *Figure S1*).

Importantly, the correlation between eGFR and serum bicarbonate levels in the study cohort was weak (Pearson r = 0.211, Supplementary material online, *Figure S2*), suggesting that serum bicarbonate may function as an independent marker of diuretic responsiveness, distinct from renal function. Notably, all decongestion markers exhibited trajectories consistent with clinical improvement, with values trending below the clinical equilibrium point.

### Safety outcomes

No substantial differences in adverse events (AEs) were observed across eGFR groups (*Table 2*). There was no significant difference in WRF events when defined as either creatinine elevation >0.3 mg/dL (18% vs. 20%) or >0.5 mg/dL (11% vs. 7.2%) from baseline or as a decline of >30% in eGFR from baseline (4.5% vs. 5.8%). Furthermore, there were no differences in incidence of hyponatremia (6.8% vs. 8.7%) or dyskalaemia (9.3% vs. 2.9%). There was one symptomatic hypotension event in the eGFR ≤30 mL/min/1.73 m^2^ group and none in the eGFR >30 mL/min/1.73 m^2^ group. There were no events of metabolic acidosis requiring HCO3 supplements in both groups.

**Table 2:** Adverse events and safety outcomes by eGFR group

Characteristic	eGFR Group (mL/min/1.73 m^2^)		*P*-value*^2^*
	≥30*^1^ (n* = *44)*	>30*^1^ (n* = *69)*	
WRF >0.3 mg/dL, no. of events, (%)	8/44(18%)	14/69(20%)	0.78
WRF >0.5 mg/dL, no. of events, (%)	5/44(11%)	5/69(7.2%)	0.46
WRF >30% eGFR decline, no. of events (%)	2/44(4.5%)	4/69(5.8%)	0.77
Hyponatraemia, no. of events, (%)	3/44(6.8%)	6/69(8.7%)	0.72
Dyskalaemia, no. of events (%)	4/43(9.3%)	2/69(2.9%)	0.72
Symptomatic hypotension, no. of events (%)	1/44(2.3%)	0/69(0%)	0.17
Metabolic acidosis requiring HCO3 infusion, no. of events	0/44(0%)	0/69(0%)	

^1^n/*n*(%).

^2^Likelihood ratio test for Generalized Linear Mixed Model (GLMM).

Abbreviations: eGFR, estimated glomerular filtration rate; WRF, worsening renal function.

## Discussion

**Figure 5:**
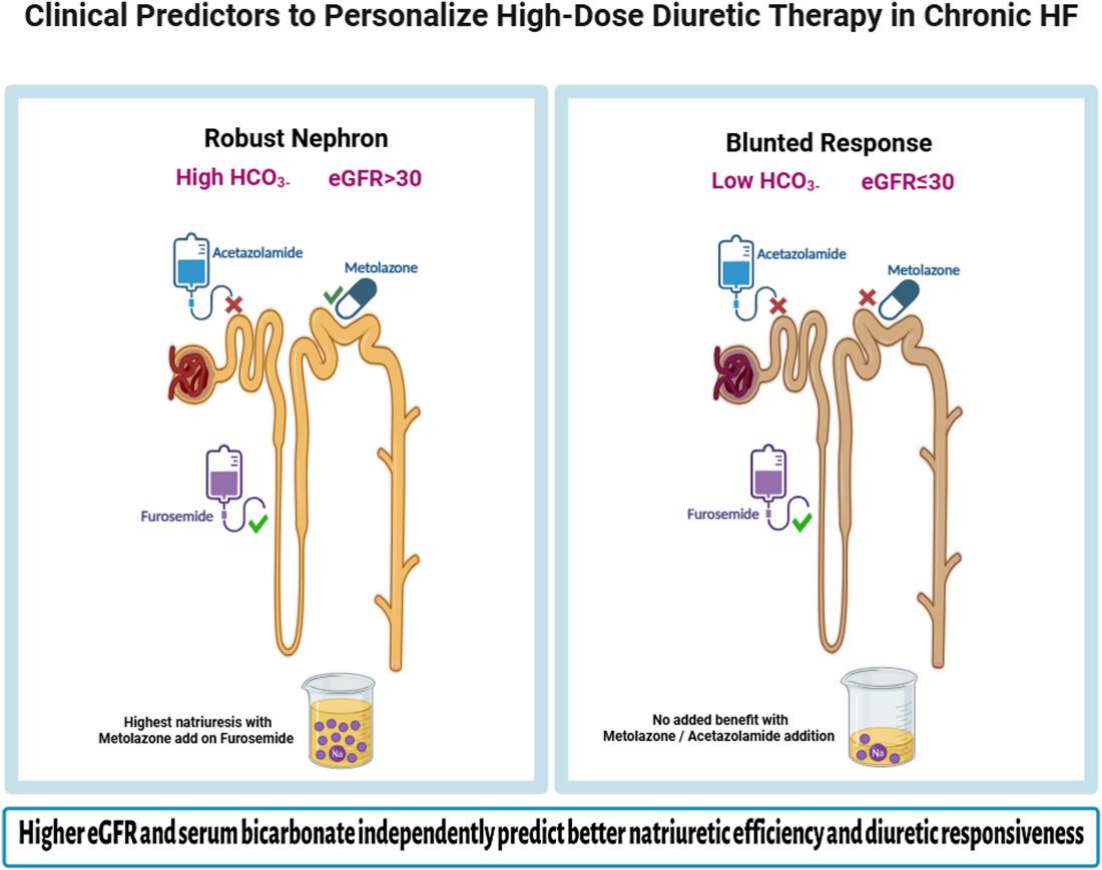
Central illustration: clinical predictors to personalize high-dose diuretic therapy in chronic HF. Renal function and acid–base status independently dictate diuretic efficacy. Patients with higher eGFR (>30 mL/min/1.73 m^2^) or a higher serum bicarbonate level exhibit a ‘robust nephron’ response, showing significant benefit from adding metolazone to furosemide. Conversely, those with lower eGFR (≤30 mL/min/1.73 m^2^) or lower serum bicarbonate level demonstrate a ‘blunted response’ with no incremental benefit from sequential nephron blockade. Ultimately, higher eGFR and serum bicarbonate independently predict better natriuretic efficiency and diuretic responsiveness.

This study, based on the DEA-HF prospective randomized trial,^1^ aimed to evaluate the efficacy of commonly used diuretic regimens across varying levels of renal function and serum bicarbonate levels in patients with chronic HF and refractory congestion. In the analysis, we found that patients with eGFR >30 mL/min/1.73 m^2^ experience a significantly higher natriuretic and diuretic response compared to those with eGFR ≤ 30 mL/min/1.73 m^2^. Among patients with eGFR >30 mL/min/1.73 m^2^, the highest natriuresis was observed when metolazone was added to furosemide, whereas among patients with eGFR ≤ 30 mL/min/1.73 m^2^, neither metolazone nor acetazolamide, when added to high-dose IV furosemide, augmented natriuresis or diuresis. Pretreatment serum bicarbonate above the median level (>29.6 mmol/L) was associated with greater natriuresis and diuresis across the three treatment groups, compared with patients with lower than median pretreatment bicarbonate level (≤29.6 mmol/L). Main findings are presented in the central illustration - *Figure 5.*

More than half of patients with chronic HF present with concomitant CKD.^4,5^ In this high-risk population, diuretic responsiveness is frequently impaired,^22^ which poses a major challenge in achieving and maintaining effective decongestion. CKD is one of the most prevalent and powerful predictors of poor outcomes in patients with HF, being strongly associated with increased risks of hospitalizations and mortality.^23,24^ This poor prognosis is closely linked to impaired natriuretic efficiency, leading to clinical diuretic resistance. Mechanistically, diuretic resistance in this setting arises from multiple intrarenal adaptive processes, including augmented proximal tubular sodium reabsorption, activation of the RAAS, and distal tubular hypertrophy, a constellation often referred to as the *diuretic braking phenomenon*.^25^ To counteract these maladaptive responses, bail-out strategies are commonly suggested to induce sequential nephron blockade, targeting sodium reabsorption both proximal to the loop of Henle (e.g. SGLT2i, acetazolamide) and distal to it (e.g. thiazide or thiazide-like diuretics, mineralocorticoid receptor antagonists, vasopressin antagonists).^26^ Although such diuretics combinations are routinely employed in clinical practice for this population, robust evidence and clear guidance on tailoring therapy for congestion-refractory HF with CKD remain limited. Understanding how renal function shapes the response to decongestive therapy in this setting is therefore essential to optimize individualized care in this complex, high-risk group.

In the current study, patients with eGFR > 30 mL/min/1.73 m^2^ exhibited significantly enhanced natriuretic and diuretic responses to diuretic therapy. This observation reiterates previous findings demonstrating the critical role of renal function in the effectiveness of diuretics and suggest that sequential distal nephron blockade can confer additive benefit, in selected patients with adequate renal reserve.^7,8,27,28^ By contrast, among patients with eGFR ≤ 30 mL/min/1.73 m^2^, both natriuresis and diuresis were significantly attenuated compared to patients with higher eGFR, and the expected potentiation form combination therapy with furosemide plus acetazolamide or metolazone was not observed. This finding contrast with data form acute decompensated HF, where acetazolamide demonstrated proportionally greater benefit in patients with reduced eGFR.^12^ Potential explanations for this discrepancy include differences in neurohormonal activation between acute vs. chronic HF, widespread use of SGLT2i and higher baseline loop diuretic doses in the chronic HF cohort (DEA-HF), and the relatively high furosemide dosing applied in this setting compared with the lower standardized doses used in ADVOR trial.^12^ With respect to thiazide therapy, our findings parallel those of the CLOROTIC post-hoc analysis, in which hydrochlorothiazide enhanced loop diuretic response across the eGFR spectrum but demonstrated a progressive decline in decongestive efficacy with WRF.^19^ Collectively, these observations may suggest a ‘ceiling effect’ in the capacity for tubular sodium handling once renal impairment surpasses a certain threshold. Mechanistically, this may reflect a reduction in functional nephron mass, alterations in tubular transporter expression, or impaired drug delivery due to reduced renal perfusion. The high prevalence of background SGLT2 inhibitor and mineralocorticoid receptor antagonist therapy in this cohort may also have influenced tubular sodium handling and baseline natriuretic tone and should be considered when interpreting the observed treatment responses.

In the DEA-HF trial, weekly high-dose diuretic therapy resulted in significant decongestion following sequential therapy for 3 weeks regardless of regimen type or eGFR.^3^ In the current study, although patients with higher eGFR had better natriuresis and diuresis, low vs. high eGFR did not differentiate the overall decongestion effect at 1 week, as assessed by reductions in body weight, serum NT-proBNP levels, LUS B-lines, and composite congestion score. This dissociation may be explained either by the relatively small sample size that may have limited the study’s ability to detect subtle differences between the groups or by the possibility that additional treatment courses are required to demonstrate measurable changes in clinical markers, as observed in the DEA-HF sub-analysis evaluating consecutive weekly treatments.^3^ In addition, 6-hour natriuresis reflects the acute pharmacodynamic response to a given regimen, whereas clinical congestion parameters assessed at 1 week represent integrated and cumulative physiological effects influenced by background therapy, dietary intake, neurohormonal activation, and repeated treatment exposure. Furthermore, biomarkers and clinical endpoints were evaluated at a single predefined time point, which may not fully capture earlier peak responses or delayed treatment effects.

Elevated serum bicarbonate levels have long been recognized as a biochemical correlate of diuretic resistance and heightened neurohormonal activation.^29^ It is also notable that bicarbonate levels tend to rise in the context of chronic loop diuretic therapy, particularly in the setting of volume contraction.^29^ In the setting of acute decompensated HF, recent evidence demonstrated that patients with higher baseline bicarbonate derived greater decongestive benefit from acetazolamide plus furosemide vs. furosemide alone, presumably by targeting proximal tubular NaHCO_3_ reabsorption and counteracting the effect of neurohormonal activation. In the current study, a consistent association between baseline serum bicarbonate levels and diuretic efficacy in patients with chronic HF was identified. Notably, among individuals with higher bicarbonate levels, furosemide plus metolazone produced superior natriuretic efficiency and diuretic responsiveness compared with either furosemide monotherapy or furosemide combined with acetazolamide. This finding may appear paradoxical, given that bicarbonate handling occurs primarily in the proximal tubule,^30^ the site of action of acetazolamide. This observation may reflect the differential pathophysiology of sodium retention in chronic vs. acute HF. Although acute HF appears more strongly influenced by proximal tubular sodium-bicarbonate reabsorption and neurohormonal activation, chronic HF with sustained loop diuretic exposure may be characterized by adaptive distal tubular hypertrophy and upregulation of sodium transporters, a phenomenon often described as the ‘diuretic braking’ effect.^7,31^ In this setting, distal sodium reabsorption may become the dominant determinant of net natriuretic response. This could explain why distal inhibition with metolazone, rather than proximal carbonic anhydrase inhibition with acetazolamide, conferred incremental benefit in patients with elevated bicarbonate levels. Thus, metolazone may exert a more sustained decongestive effect in chronic HF, overcoming resistance mechanisms that extend beyond proximal tubular bicarbonate handling. Additionally, differences in clinical context and patient characteristics may account for the observed divergence from ADVOR as outlined above. Importantly, in this analysis the correlation between eGFR and bicarbonate was weak (Pearson r = 0.21), indicating that the predictive value of bicarbonate is largely independent of baseline renal function. This reinforces its potential utility as a biochemical marker of diuretic responsiveness across the HF spectrum, including in patients with advanced CKD, in whom diuretic resistance is most prevalent.^4,25^ Hence, serum bicarbonate levels could serve as a simple, readily available biomarker to predict diuretic response in patients with chronic HF, including those with advanced CKD (eGFR ≤30 mL/min/1.73 m^2^). Such a marker could further help tailor diuretic strategies, guiding decision-making in diuretic therapy such as therapy escalation or considering alternative decongestive interventions such as ultrafiltration.

No significant differences in AEs were observed between patients with eGFR >30 mL/min/1.73 m^2^ and those with eGFR ≤30 mL/min/1.73 m^2^. WRF was rare across both groups. Rates of hypotension, dyskalaemia, and hyponatremia were similarly low and did not differ meaningfully between strata. These findings are particularly notable, given the use of high-intensity diuretic therapy, and collectively support the renal and electrolyte safety of this approach, even in patients with advanced renal impairment.

The findings of this study are particularly relevant, considering the growing population of HF patients with coexisting CKD. These individuals often fall outside the scope of large clinical trials and are at increased risk for AEs, including hospitalization and mortality.^24^ The findings of this study provide clinically relevant insights for this underserved population. By elucidating the interplay between renal function, serum bicarbonate, and decongestive response, these results may inform the development of more nuanced therapeutic strategies, enabling treatment to be more precisely tailored to the individual patient’s physiological profile. These findings apply to a clinically stable, ambulatory chronic HF population with refractory congestion and should be interpreted within this context.

Several limitations warrant consideration. This is a single-center, open-label study with a relatively small cohort that extracted its statistical power from crossover design. The sub-analyses were exploratory in nature and underpowered for the subgroup comparisons. The absence of an observed incremental benefit of combination therapy in patients with advanced renal impairment should therefore be interpreted with caution, given the relatively small sample size and limited statistical power of this subgroup analysis. Biomarkers and clinical endpoints were assessed at a single time point 1 week following intervention, which may have missed either delayed or cumulative effects from multiple treatments or, alternatively, may have failed to capture peak therapeutic responses occurring earlier in the week. In addition, more direct assessments of fluid overload, such as inferior vena cava measurements or bioimpedance analysis, were not performed and may have provided complementary information regarding volume status. Furthermore, metolazone was administered at a fixed dose of 5 mg per protocol; whether higher doses would yield greater natriuretic response in patients with advanced renal dysfunction was not evaluated and warrants further study.

In conclusion, natriuretic and diuretic efficacy in patients with congestion-refractory chronic HF were positively associated with both higher eGFR and elevated serum bicarbonate levels, regardless of the diuretic regimen administered. While natriuretic response was clearly influenced by renal function, effective decongestion was achieved across all eGFR strata. Notably, in patients with either eGFR >30 mL/min/1.73 m^2^ or pretreatment serum bicarbonate >29.6 mmol/L, the addition of metolazone was associated with significantly improved natriuresis. However, this benefit was not evident in patients with lower eGFR or lower serum bicarbonate levels, suggesting that these two factors each contribute to diuretic responsiveness and may help identify those most likely to benefit from combination diuretic strategies.

## Supplementary Material

pvag019_Supplementary_Data

## Data Availability

The data underlying this article will be shared on reasonable request to the corresponding author.
