# Supplementary figures and images for: Kidney function and acid–base status as gatekeepers of natriuretic and diuretic responsiveness in chronic HF: insights from the DEA-HF clinical trial
Source: Eur Heart J Cardiovasc Pharmacother. 2026 May 18;12(4):309–19. doi: 10.1093/ehjcvp/pvag019 (PMC13367239; doi:10.1093/ehjcvp/pvag019)

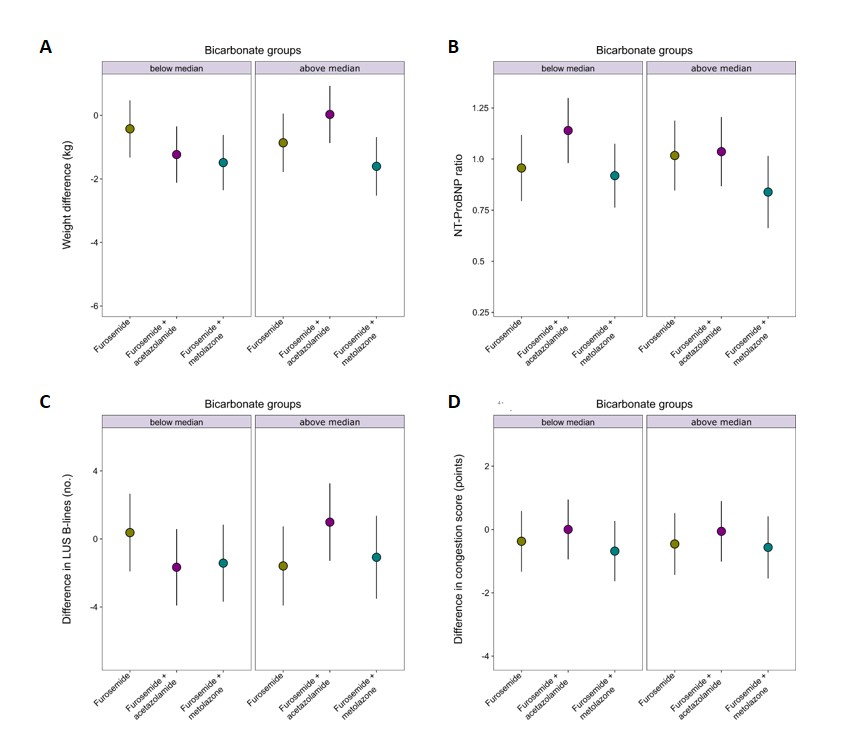

Supplement: pvag019_Supplementary_Data [file pvag019_supplementary_data.zip › Sup. Figure 1.jpg]

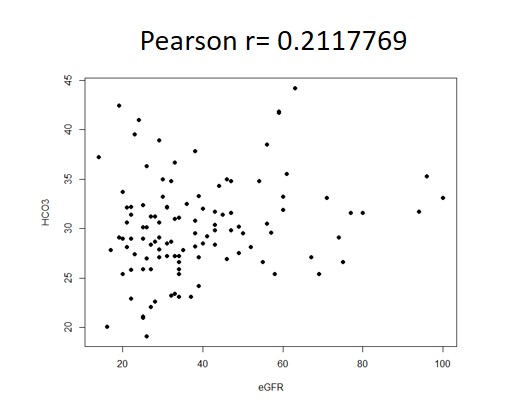

Supplement: pvag019_Supplementary_Data [file pvag019_supplementary_data.zip › Sup. Figure 2.jpg]

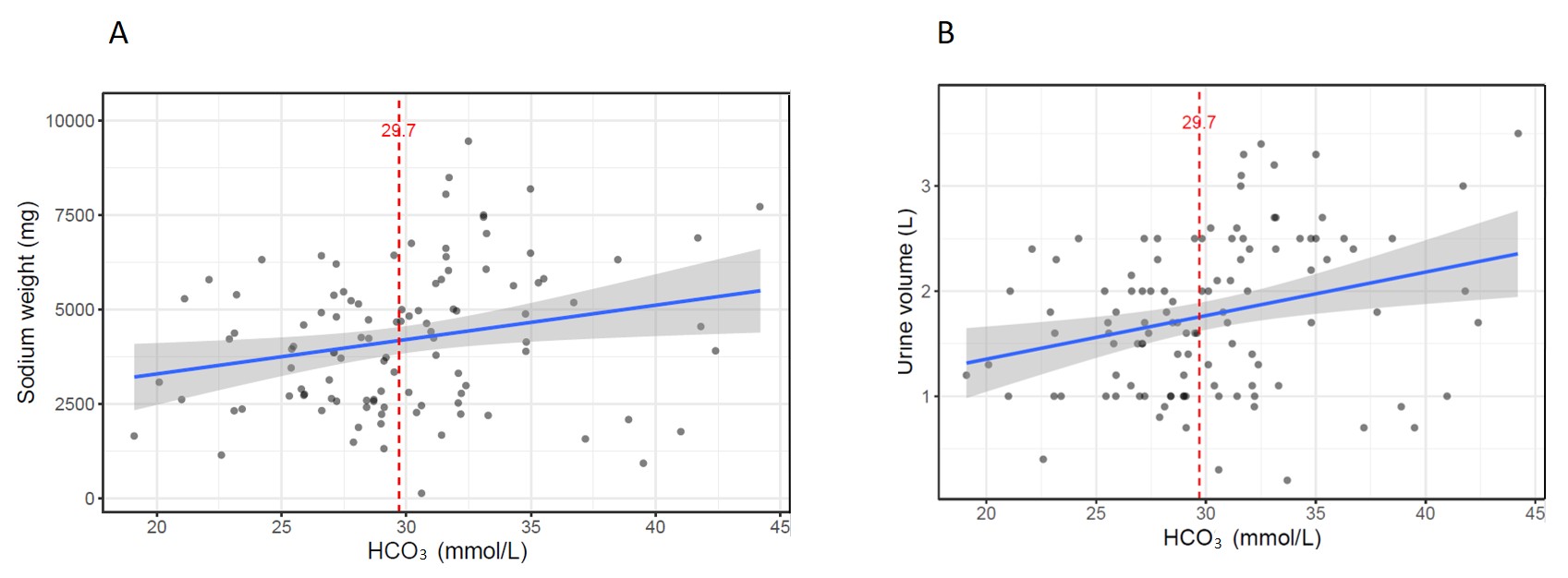

Supplement: pvag019_Supplementary_Data [file pvag019_supplementary_data.zip › Sup. Figure 3.jpg]
